# Tunable Self-Assembled Nanostructures of Electroactive PEGylated Tetra(Aniline) Based ABA Triblock Structures in Aqueous Medium

**DOI:** 10.3389/fchem.2019.00518

**Published:** 2019-07-25

**Authors:** Irrum Mushtaq, Zareen Akhter, Faiz Ullah Shah

**Affiliations:** ^1^Department of Chemistry, Quaid-i-Azam University, Islamabad, Pakistan; ^2^Chemistry of Interfaces, Luleå University of Technology, Luleå, Sweden

**Keywords:** triblock nanostructures, PEGylation, redox activity, aqueous self-assembly, tunable self-assembled structures

## Abstract

PEGylated tetra(aniline) ABA triblock structure PEG-TANI-PEG (**2**) consisting of tetra(aniline) (TANI) and polyethylene glycol (PEG) was synthesized by coupling the tosylated-PEG to boc-protected NH_2_/NH_2_ TANI (**1**) through a simple nucleophilic substitution reaction. Deprotection of **2** resulted in a leucoemeraldine base state of TANI (**2**-LEB), which was oxidized to stable emeraldine base (**2**-EB) state. **2**-EB was doped with 1 M HCl to emeraldine salt (**2**-ES) state. FTIR, ^1^H and ^13^C NMR and UV-Vis-NIR spectroscopy, and MS (ESI) was used for structural characterization. The synthesized triblock structure exhibited good electroactivity as confirmed by CV and UV-Vis-NIR spectroscopy. Self-assembling of the triblock structure in aqueous medium was assessed by DLS, TEM, and SEM. Spherical aggregates were observed with variable sizes depicting the effect of concentration and oxidation of **2**-LEB. Further, the aggregates showed acid/base sensitivity as evaluated by doping and dedoping of **2**-EB with 1 M HCl and 1 M NH_4_OH, respectively. Future applications in drug delivery and sensors are envisaged for such tunable self-assembled nanostructures in aqueous media.

## Introduction

Self-assembly is the simplest approach toward the production of highly ordered functional assemblies of molecules in a wide range of interesting morphologies (e.g., various micellar structures, nanorods, nanoribbons, nanofibers, amongst others) (Gröschel and Müller, 2015; Townsend et al., 2018). Such structures have potential applications in the field of nanotechnology, biomedicine, tissue engineering and drug delivery (Kutikov and Song, 2015; Li et al., 2017; Zhou et al., 2017; Park et al., 2018; Liua et al., 2019). This self-assembly approach produces such intricate morphologies through various secondary interactions (Molla and Ghosh, 2014), including π-π stacking (Herrikhuyzen et al., 2006), hydrogen bonding (Valkama et al., 2006), ionic interactions (Faul, 2014) and hydrophobic interactions (Lombardo et al., 2015). The presence of solvent plays a key role in determining the morphology of the material, and can be used to tune final morphologies (Shao et al., 2012; Echue et al., 2015; Dane et al., 2016). In aqueous self-assembly, the interesting properties of water play an important role in the formation of defined structures owing to its non-covalent interactions, leading to its unique potential for biomedical applications (Sun et al., 2017).

Recently, a significant interest in the aqueous self-assembly of conjugated polymers and related lower molecular weight analogs has been observed (Magnieto et al., 2017; Zhang et al., 2019). Due to the very strong stacking interactions and the hydrophobic nature of such conjugated materials, their solubility in aqueous media is very low. In order to address these issues, various approaches have been tried to enhance the solubility of polymeric and oligomeric conjugated structures. Some examples of such approaches are chemical modification of conjugated poly- and oligoelectrolytes (Dey et al., 2018), conjugation with peptides and other water-soluble biomolecules (Jatsch et al., 2010; Diaferia et al., 2016) and PEGylation i.e., the introduction of amphiphilicity by incorporating hydrophillic polyethylene glycol polymer chains (Hamley, 2014). In the case of the well-studied conducting polyaniline (PANI) polymer and its lower oligomers, such investigations are not very well-known.

We focus our efforts on the shortest functional oligomer of PANI, tetra(aniline), or TANI. It exhibits the same unique redox activity and electroactivity, as the parent polymeric analog PANI (Wei and Faul, 2008): TANI has three oxidation states, a colorless or gray fully reduced leucoemeraldine base (LEB) state, a blue half-oxidized emeraldine base (EB) state and a dark, deep-violet color fully oxidized pernigraniline base (PB) state. When the EB state is doped with an acid it is converted to the conducting green emeraldine salt (ES) state. Owing to the existence of these interesting redox states, switchability can be induced in this class of materials which can be exploited in many interesting applications (Udeh et al., 2011; Hu et al., 2017). PANI's limited solubility and processability can be overcome by the introduction of TANI, owing to its good solubility in common organic solvents (and thus processability) and defined molecular structure (Udeh et al., 2011). TANI-based materials self-assemble into defined structures due to its rigid aromatic structure and strong stacking and hydrophobic interactions (Zhao et al., 2013; Lyu et al., 2018). Studies have also shown that this electroactive motif can also be used to introduce and use an “addressable packing parameter” to tune self-assembly behavior in simple single-tailed cationic TANI-based amphiphiles (Lyu et al., 2018). In various amphiphilic block-copolymers (with various hydrophilic blocks) TANI has been included to produce amphiphilic electroactive structures. However, their greater hydrophillicity to hydrobicity ratio make these systems fairly soluble on doping with acids or aggregates of undefined sizes (Huang et al., 2007; Hu et al., 2008). Moreover, in most of the TANI based amphiphiles amide linkage is introduced between TANI and hydrophillic moiety (Yang et al., 2010; Kim et al., 2011; Bell et al., 2015; Dong et al., 2015). None of these systems was investigated by changing the linkage between TANI and hydrophillic block. We thus made an attempt to synthesize such a low molecular weight amphiphiles in which TANI was connected with hydrophillic block (PEG) through secondary amine linkage. The hydrophobicity to hydrophilicity ratio was controlled by using the short chain length mPEG_350_ (*n* = 8) leading to self-assembled structures of defined size in neutral, acidic and basic aqueous media.

In this study, we successfully synthesized low molecular weight ABA amphiphilic triblock structures consisting of PEGylated-TANI (**2**). Briefly TANI was coupled with octa(ethylene glycol) methyl ether (mPEG_350_) forming secondary amine (NH) linkage by a simple nucleophilic substitution reaction. Deprotection of **2** resulted in the formation of **2**-LEB which was oxidized to **2**-EB followed by doping with HCl to **2**-ES. The electroactive properties of the PEGylated TANI in different oxidation states (**2**-LEB, **2**-EB, and **2**-ES) were studied by ultraviolet-visible-near infrared (UV-Vis-NIR) spectroscopy and cyclic voltammetry (CV). Aqueous self-assembly was studied by using different microscopic techniques including TEM, SEM, and DLS. **2**-LEB self-assembles into spherical aggregates, which are switched to larger sized aggregates on increasing concentration. On changing oxidation state of **2**-LEB to the half-oxidized state (**2**-EB), the size of the spherical aggregates was reduced. Further the self-assembly behavior of the material was investigated by doping of **2**-EB with an acid (1 M HCl, **2**-ES) and dedoping by adding a base (1 M NH_4_OH). This doping/dedoping process showed a change in size of the aggregates without disturbing the spherical morphology of the aggregates forecasting the application in controlled drug delivery and sensors.

## Experimental Section

### Materials

Monomethoxy octa(ethylene glycol) with a number average molecular weight of 350 (mPEG_350_) (obtained from Aldrich) was azeotropically distilled with toluene prior to use. Ammonium persulphate (APS), dimethylaminopyridine (DMAP) and triethylamine were purchased from Aldrich and used as received. Dimethylsulfoxide (DMSO) and trifluoroacetic acid (TFA) of reagent grade were obtained from Merck. Dried tetrahydrofuran (THF), diethyl ether and dichloromethane (DCM) were obtained from an Anhydrous Engineering dry solvent system based on the Grubbs' design (Pangborn et al., 1996).

### Methods

Fourier transform infrared (FTIR) spectra were recorded on Perkin Elmer spectrum 100 FTIR spectrophotometer. ^1^H and ^13^C nuclear magnetic resonance (NMR) spectra were obtained from VNMRS400 spectrometer in deuterated chloroform (CDCl_3_) and dimethyl sulfoxide-d6 (DMSO-*d6*) as solvents. Perkin Elmer Lambda 35 UV-Visible spectrophotometer was used to record the solution-state UV-Vis spectra of TANI-based materials. Cyclic voltammetry (CV) was performed on Gamry potentiostat/galvanostat interface 1000 in 1.0 M H_2_SO_4_ aqueous solution using Ag/AgCl as the reference electrode and Pt wire as the counter electrode. The working electrode was fluorine doped tin oxide (FTO) glass coated with sample aqeous solution. The cyclic voltammograms were measured in the range from −0.1 to 1.0 V at different scan rates of 50–150 mV/s. Critical micelle concentration (CMC) was determined by Perkin Elmer Lambda 35 UV/VIS spectrophotometer. Absorption spectra of the solutions were recorded by varying the concentration from 0.001 to 0.1 mM in water. Transmission electron microscopy (TEM) images were observed using a JEOL JEM 1200 EX microscope at an accelerating voltage of 120 kV. Samples were drop-cast onto copper TEM grids coated with carbon film. Dynamic light scattering (DLS) measurements were carried out to determine the particle size distribution using a Malvern Zeta Sizer Nano series equipped with a laser of a wavelength of 633 nm and a detector oriented 173° to the incident radiation. Samples of different concentrations were prepared in deionized water and filtered through 0.45 μm nylon syringe filter prior the measurement. Autocorrelation function (ACF) was obtained from DLS data. Scanning electron microscopy (SEM) images were taken on TESCAN Vega LUM equipment. A drop of micellar solution in water was drop casted on glass surface at room temperature for SEM assessment.

### Synthesis

#### Synthesis of NH_2_/NH_2_ Boc-Protected Tetra(Aniline) TANI (1)

NH_2_/NH_2_ boc-protected tetra(aniline) TANI (**1**) was synthesized in three steps. NO_2_/NO_2_ boc-protected TANI was synthesized by the reported method in two steps (Eelkema and Anderson, 2008). The reduction of NO_2_/NO_2_ boc-protected TANI was carried out using ammonium formate in the presence of activated Pd on charcoal to yield the desired product **1** (Ram and Ehrenkaufer, 1984). Yield: 91%. ^1^H NMR (400 MHz, CDCl_3_, δ ppm): 7.10 (d, *J* = 4.0 Hz, 4H, Ar-N(boc)-Ar), 7.05 (d, *J* = 4.0 Hz, 4H, Ar-N(boc)-Ar), 6.81 (d, *J* = 8.0 Hz, 4H, Ar-N-boc), 6.50 (d, *J* = 8.0 Hz, 4H, Ar-NH2), 5.10 (br. s, 4H, Ar-N*H2*), 1.32 (d, *J* = 4.0 Hz, 27H, boc(*t*-C*H3*); ^13^C NMR (101 MHz, CDCl_3_, δ ppm):152 (Boc C = O), 139 (Ar-NH_2_), 128 (Ar-N(boc)-Ar), 126 (Ar-N(boc)-Ar), 115 (NH_2_-Ar), 81 (boc (C(CH)_3_), 28 (boc(t-CH_3_)); FTIR (solid sample, cm^−1^) ν: 3,467 (m, ν NH), 3,360 (m, ν NH), 2,925 (m, ν sp^3^-CH), 1,722 (s, ν boc C = O), 1,510 (s, ν C = C benzenoid), 1,061 (s, ν boc C-O-C). MS (ESI) m/z: 704.3 [M + Na]^+^.

#### Tosylation of mPEG_350_

mPEG_350_ was tosylated by using a reported method (Chatterjee and Ramakrishnan, 2014). Briefly in a three necked round bottom flask, mPEG_350_ (15.5 mmol, 5.44 g) was taken in 5 mL THF and the contents were cooled in an ice bath. Sodium hydroxide (25 mmol, 1 g, 20% aq. solution) was added in the cooled mixture followed by slow addition of p-toluenesulfonyl chloride (14.4 mmol, 2.74 g) in 5 mL of THF using a dropping funnel over a period of 2 h. The solution was stirred at 0–5°C for an additional 2 h and then poured into ice cold water (20 mL). Organics were extracted with chloroform twice. The combined organic extracts were washed with water twice than with brine once and then dried over anhydrous MgSO_4_. The solvent was evaporated resulting tosylate as a white solid. Yield: 90%. ^1^H NMR (400 MHz, CDCl_3_, δ ppm): 7.77 (d, *J* = 8 Hz, 2H, Ar-SO_2_), 7.33 (d, *J* = 8 Hz, 2H, CH_3_-Ar), 4.14 (t, *J* = 8 Hz, 2 H, SO_2_-OC*H*_2_), 3.66–3.56 (m, 27H, C*H*_2_-C*H*_2_-O), 3.36 (s, 3H, OC*H*_3_), 2.43(s, 3H, Ar-C*H*_3_); ^13^C NMR (101 MHz, CDCl_3_, δ ppm): 140 (Ar-SO_2_), 130 (Ar), 71 (CH_2_-CH_2_-O), 68 (SO_2_-OCH_2_), 58 (OCH_3_), 25 (Ar-CH_3_); MS (ESI) m/z: 561.2 [M + Na]^+^.

#### Synthesis of PEGylated Tetra(Aniline) ABA Type Triblock Structure (PEG)_8_-TANI-(PEG)_8_ (2)

**1** (1 eq., 0.73 mmol, 500 mg), tosylate-mPEG_350_ (2.1 eq., 1.53 mmol, 0.8 mL) and DMAP (52 mg) were placed in a three-necked flask and protected under nitrogen. DMSO (5 mL) was added followed by triethylamine (1 mL) and stirred at 65 °C overnight. The reaction mixture was cooled and precipitated in diethyl ether. The precipitate was dissolved in THF and reprecipitated by n-hexane. This dissolution/precipitation process was repeated three times. Then precipitate obtained was dissolved in ethyl acetate, reprecipitated with n-hexane three times and dried under vacuum overnight. Yield: 40%. ^1^H NMR (400 MHz, CDCl_3_, δ ppm): 7.09 (m, 8H, (boc)N-Ar-N(boc)), 6.89 (d, *J* = 8 Hz, 4H, (boc)N-Ar-NH-), 6.53 (d, *J* = 8 Hz, 4H, NH-Ar), 3.65 (m, 54H, O-C*H*_2_-C*H*_2_-O), 3.38 (s, 6H, OCH_3_), 3.23 (t, *J* = 8 Hz, 4H, NH-C*H*_2_), 1.42 (s, 27H, boc *t*-C*H*_3_). ^13^C NMR (101 MHz, CDCl_3_, δ ppm): 144 (boc C = O), 139 (Ar-C-NH), 128 (Ar-N(boc)), 126 (Ar), 71 (O-CH_2_-CH_2_-O), 61 (OCH_3_), 46 (HN-CH_2_), 32 (boc *t*-CH_3_); FTIR (solid sample, cm^−1^) ν: 3,364 (m, ν NH), 3,045 (w, ν C = C), 2,979 (s, ν CH_3_), 2,869 (s, ν CH_2_), 1,696 (s, ν boc C = O), 1,603 (m, ν NH) 1,510 (s, ν C = C benzenoid), 1,091 (m, ν C-O-C); MS (MALDI-TOF) m/z: 1,381.

#### Deprotection of 2 (2-LEB)

Boc-protected product **2** was dissolved in anhydrous DCM and cooled to 0°C. TFA (equal amount as the solvent) was added and the solution allowed to warm to room temperature. After stirring at room temperature until all starting material was consumed (as checked by TLC), the solution was concentrated under reduced pressure. The solution was extracted with distilled water, saturated NaHCO_3_, water and brine, three times (3 × 10 mL). The combined organic fractions were dried over MgSO_4_, filtered, evaporated under reduced pressure and dried under vacuum overnight. A dark gray-colored product was obtained. Yield: 50%. ^1^H NMR (400 MHz, CDCl_3_, δ ppm): 6.78 (m, 8H, Ar-N(boc)-Ar), 6.57 (d, *J* = 8 Hz, 4H, NH-Ar-N(boc)), 6.53 (d, *J* = 8 Hz, 4H, NH-Ar), 3.6–3.4 (m, 54H, O-C*H*_2_-C*H*_2_), 3.3 (s, 6H, OC*H*_3_), 3.1 (t, *J* = 8 Hz, 4H, NH-C*H*_2_), 4.8 (s, NH). UV (DMSO) λ_max_ = 321 nm (benzenoid π-π^*^).

#### Oxidation State Switching and Doping

**2-EB: 2**-LEB (1 eq., 0.04 mmol, 50 mg) was stirred in THF (10 mL) and a solution of ammonium persulphate (1 eq. 0.04 mmol, 9 mg) in 2 M HCl (10 mL) was added to the stirring solution. A dark-green solution was immediately formed. After 1 h 2 M NH_4_OH (10 mL) was added to the reaction mixture. The solution turned blue and was stirred for an additional 4 h. The reaction mixture was extracted with chloroform (3 × 10 mL). Combined chloroform layers were washed with water (3 × 5 mL) and brine (1 × 5 mL), dried over MgSO_4_, filtered and residual solvent removed and dried under vacuum overnight. Yield: 98%. UV (DMSO) λ_max_ = 305 nm (benzenoid π-π^*^), 587 nm (quinoid π-π^*^).

**2-ES: 2**-EB was dissolved in 1 M HCl and stirred overnight until UV-Vis spectroscopy showed the absence of the characteristic EB peak at 580 nm, confirming completion of the doping process. Yield: 96%. UV (DMSO) λ_max_ = 305 nm (benzenoid π-π^*^), 420 nm polaron-π^*^), 978 nm (π-polaron).

#### Preparation of Microstructures

Micellar solutions of **2** in LEB, EB and ES states were prepared by directly dissolving the predetermined amount of samples per 1 mL of deionized water (Hu et al., 2008). For doped and dedoped samples 1 M HCl and 1 M NH_4_OH were used instead of water. The solutions were kept overnight for complete self-assembly and then subjected for detailed studies.

## Results and Discussion

### Synthesis and Characterization of PEGylated Tetra(Aniline) ABA Type Coil-Rod-Coil Triblock Structure (PEG)_8_-TANI-(PEG)_8_ (2)

**Scheme 1:**
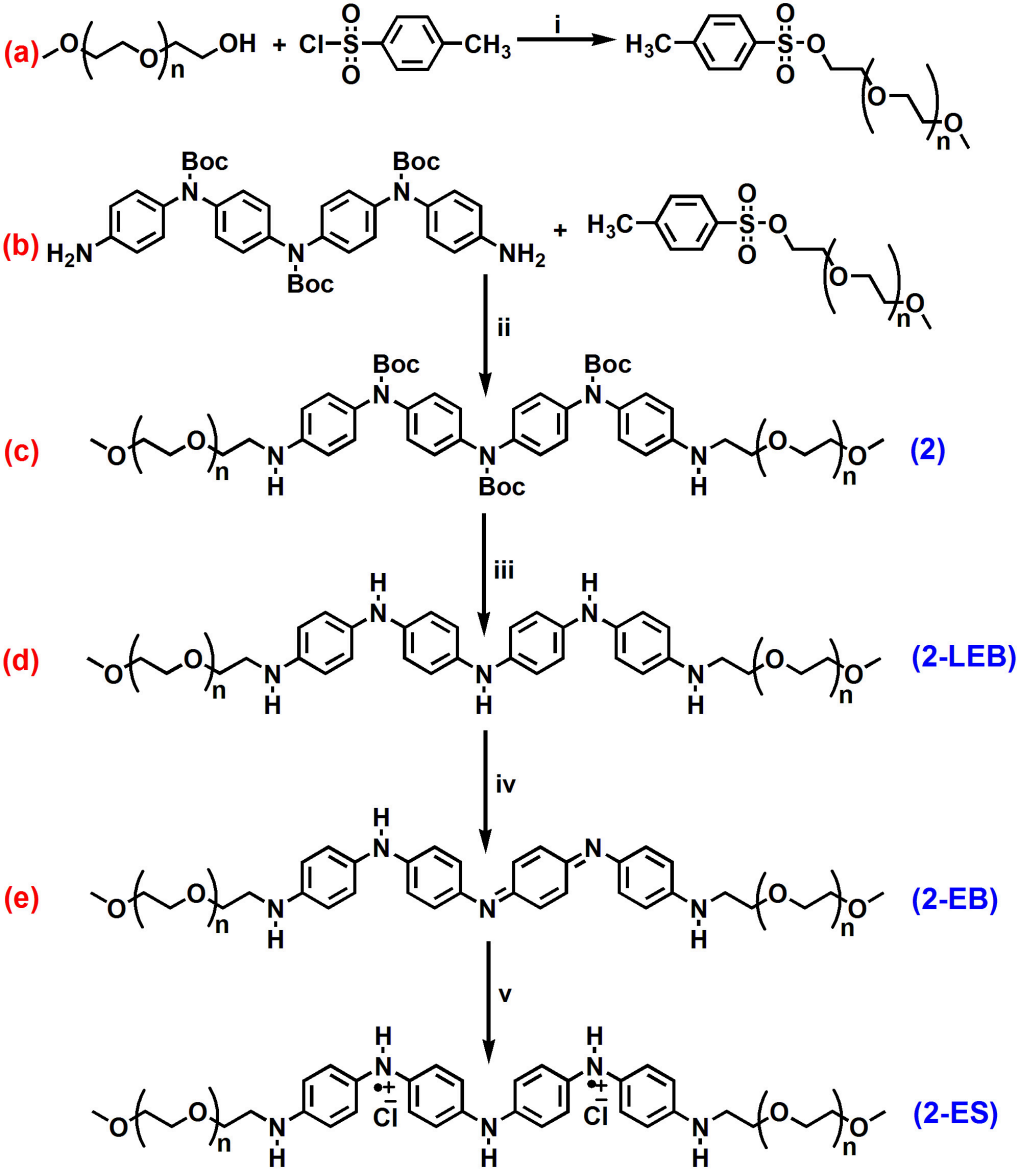
Synthesis of **(a)** tosylated-PEG, **(b)** ABA type triblock structures containing secondary amine (NH) linkage **(2)**, **(c)** deprotection, **(d)** oxidation and **(e)** doping.

**Figure 1:**
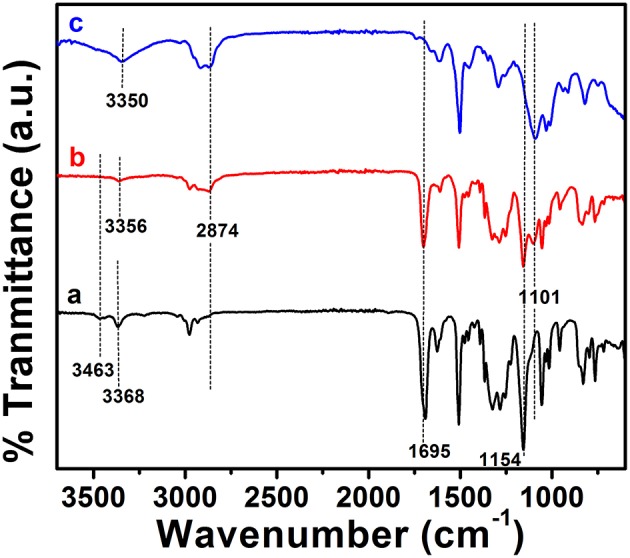
FTIR spectra of **(a) 1** (bottom trace), **(b) 2** (middle trace), and **(c) 2**-LEB (top trace). The spectra are shifted along the direction of intensity for clarity.

**Figure 2:**
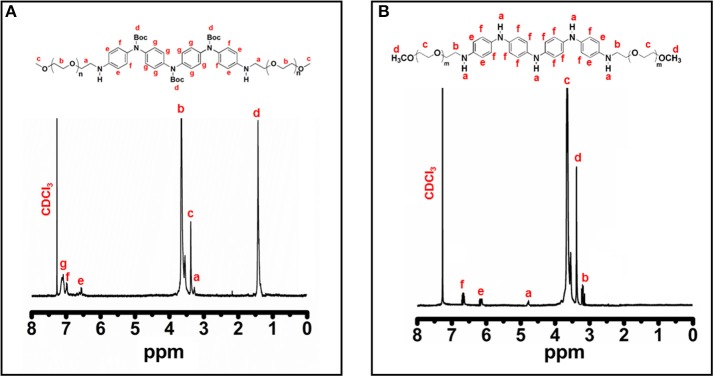
^1^H NMR spectra of **(A) 2**, and **(B) 2-**LEB in CDCl_3_.

ABA triblock structure **2** was synthesized successfully as shown in Scheme 1. The terminal hydroxyl group of the mPEG_350_ precursor is less susceptible to nucleophilic attack by an amine group; hence, tosylation of mPEG_350_ was carried out. Deprotection of the **2** resulted in the LEB state, which was further converted to the EB state by oxidation (Scheme 1). The purity and structures of the synthesized materials were elucidated by using FTIR, ^1^H NMR, and ^13^C NMR spectroscopic analyses. Figure 1 shows the FTIR spectra with annotated peaks for the different moieties present in the structures. The appearance of the stretching vibration band at 3,356 cm^−1^ for the secondary amine N-H in **2** and the disappearance of stretching bands for the primary NH_2_/NH_2_ boc-protected tetra(aniline) at 3,463 and 3,368 cm^−1^ confirmed the formation of the product (Figure 1b). The incorporation of mPEG_350_ in the product was assessed by the presence of stretching vibrations at 2,874 and 1,101 cm^−1^ for C-H and C-O-C, respectively. Deprotection of **2** resulted in the disappearance of chracterisitic peaks for boc group at 1,695 and 1,154 cm^−1^ for ester carbonyl and C-O-C moities (Figure 1c). A broad stretching vibration was appeared for NH moieties of TANI in **2-**EB at 3,350 cm^−1^. Figure 2 shows the ^1^H NMR spectra of **2** and **2**-LEB. The characteristic peaks for aromatic protons of the boc-protected TANI segment in **2** appeared at 7.09–6.53 ppm (Figure 2A). The characteristic resonance lines for mPEG_350_ segment were observed at 3.65 ppm (for methylene protons) and 3.38 ppm (for terminal methoxy protons). The disappearance of the peak at ~5.0 ppm for the NH_2_ protons of **1** confirms the formation of the product. Deprotection of **2** resulted in the conversion to fully reduced TANI segment in **2**-LEB (Figure 2B). The aromatic protons upfield to 6.78 ppm while signals at 1.42 ppm disappeared confirming the removal of boc moiety.

### Solubility

The solubilities of **1**, **1**-LEB, and **2** in both the LEB and EB states were investigated in water and common organic solvents. The results are displayed in Table 1. **1** was soluble in a selection of common organic solvents, such as chloroform (CHCl_3_), dichloromethane (CH_2_Cl_2_), tetrahydrofuran (THF), *N, N*-dimethylformamide (DMF), and dimethyl sulfoxide (DMSO). **1** in the LEB or EB state was insoluble in common organic solvents. **2** in the LEB and EB states was soluble in water as well as in the above-mentioned organic solvents, showing that solubility was increased by incorporating ethylene glycol units in the ABA block structure. Moreover, aqueous solutions of **2** in the LEB and EB state remained stable over many weeks without any precipitation.

**Table 1:** Solubility data for the studied materials in water and common organic solvents.

**Samples**	**H_**2**_O**	**CH_**2**_Cl_**2**_**	**CHCl_**3**_**	**THF**	**DMF**	**DMSO**
**1**	+	+	+	+	+	+
**1**-LEB	–	–	–	–	+	+
mPEG-350	+	+	+	+	+	+
**2**-LEB	+	+	+	+	+	+
**2**-EB	+	+	+	+	+	+

*+, soluble; –, insoluble*.

### Optoelectronic Properties

**Figure 3:**
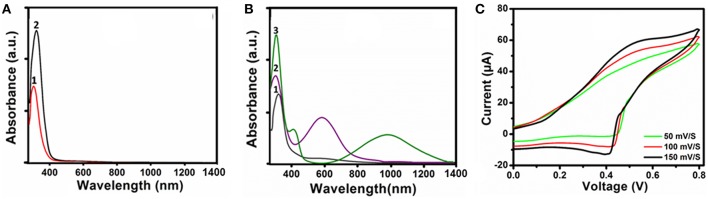
UV-Vis-NIR spectra: **(A) 1**-LEB (1) and **2**-LEB (2), **(B) 2**-LEB (1), **2**-EB (2) and **2**-ES, doped with 1M HCl (3), and **(C)** Cyclic voltammogram of **2**-EB at different scan rates in 1M H_2_SO_4_ as an electrolyte.

UV-Vis-NIR spectroscopy was used to elucidate the redox states of the TANI functional subunit in the ABA triblock structures. Figure 3 shows the UV-Vis-NIR spectra of NH_2_/NH_2_ TANI and **2** in the LEB, EB and their 1 M HCl doped ES states in water. A bathochromic shift was observed for the π-π^*^ transition from 309 to 321 nm when comparing NH_2_/NH_2_ TANI-LEB to **2**-LEB (Figure 3A). A hypsochromic shift was observed from 321 to 305 nm by oxidizing **2** from the LEB to the EB state (Figure 3B, curve 2), and the appearance of the typical peak at 587 nm was assigned to a further π-π^*^ transition for the quinoid ring structure (Figure 3B, curve 2) (Shao et al., 2011). Doping of the **2-**EB state with 1 M HCl resulted in the occurrence of two bands at 420 nm and 978 nm (Figure 3B, curve 3), typical for doped states of TANI-based materials. The appearance of the band at 420 nm is assigned to a polaron-π^*^ transition (Udeh et al., 2013). Disappearance of the absorption band at 587 nm with the appearance of the typical long wavelength π-polaron absorption band at 978 nm showed the conversion of the EB state to the conducting ES state (Lv et al., 2015). Cyclic voltammetry was performed to access the redox activity of the **2**-EB. A characteristic single reversible redox peak was obtained at a mean peak potential (E_1/2_) of 0.53 V showing the conversion of LEB to EB state (Figure 3C) (Yang et al., 2010). Peak current was increased with increasing the scan rate showing the good electrochemical reversibility (Inamdar et al., 2011). Results of UV-Vis spectroscopy and cyclic voltammetry confirmed the good electroactivity of **2**-EB.

### Aqueous Self-Assembly

#### Critical Micelle Concentration

Critical micelle concentration (CMC) of the amphiphiles was determined by using UV-Vis spectroscopy without probe (Fatima et al., 2016). UV-Vis spectra of **2**-EB in water were recorded at varying concentrations (0.001–0.1 mM) and a graph was plotted between absorbance vs. concentration (Figure S1). The graph showed two liner sections with slightly different slopes intersecting at a point denoted as CMC point. Below the intersection, point absorbance increased linearly with increasing concentration obeying the Lambert-Beer law. Above this point, deviation from the linearity upon increasing concentration indicated aggregation phenomenon (Winiewska et al., 2017). This might be due to the hurdle that is faced by UV-Vis radiations to pass through the inner core of the aggregates. **2**-EB showed very low CMC value (0.016 mM = 16 μM) depicting a very rapid balance between hydrophobic (TANI) and hydrophilic (PEG) segments toward aggregation.

#### Morphology and Size

**Figure 4:**
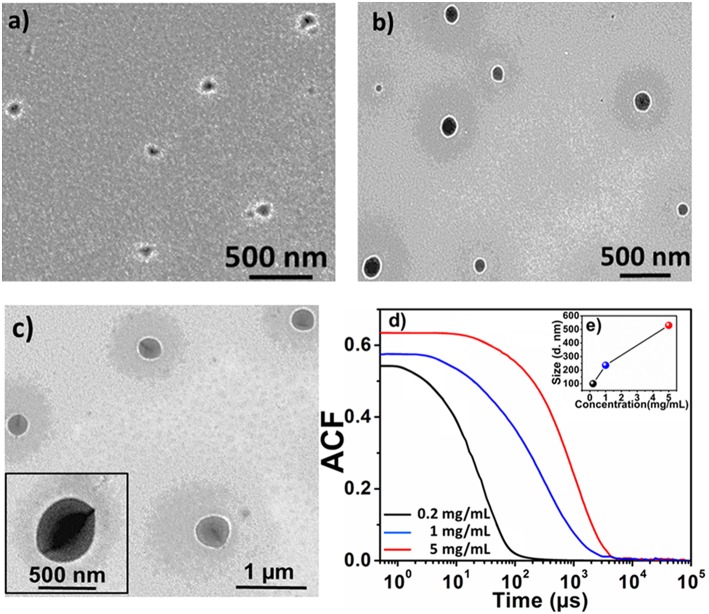
TEM images of **2-**LEB at different concentrations; **(a)** 0.2 mg/mL, **(b)** 1 mg/mL, **(c)** 5 mg/mL and DLS analysis of **2**-LEB; **(d)** Auto-correlation function (ACF), **(e)** particle size distribution at different concentrations shown as insert in **(d)**.

For aqueous self-assembly of these non-ionic amphiphilic materials, sample solutions were prepared by directly dissolving the sample in double distilled water (Hu et al., 2008). Samples of **2** in the LEB, EB and 1 M HCl doped ES states, were prepared to study the effect of concentration, oxidation state and doping on size and morphology of the formed aggregates, respectively. Figure 4 shows the morphology and size of the **2-**LEB aggregates formed in aqueous solution at different concentrations. It was observed that increase in the concentration lead to increase in the size of the aggregates. At 0.2 mg/mL, triblock structures self-assembled into spherical aggregates of 80 nm size as observed by TEM (Figure 4a). Upon increasing concentration from 1 to 5 mg/mL, spherical objects of larger size in the range from 230 to 500 nm were observed by TEM (Figures 4b,c). DLS studies also showed the presence of similar size objects in the aqueous solution with a size difference of ±10 nm from TEM (Figure S2). This may be attributed to the drying effect resulting in shrinkage of size of the objects on evaporation of the solvent (Lyu et al., 2018). Further DLS-derived auto-correlation function (ACF) also demonstrated the increase in the size of the aggregates with increasing concentration (Figures 4d,e). Monomodal distribution of the aggregates was observed for each concentration showing the uniformity of the aggregate formation. Larger size aggregates showed less relaxation time due to their decreased diffusion velocity as compared to smaller size aggregates, obeying Stokes-Einstein equation, which shows inverse relation between hydrodynamic radius and diffusion velocity (Winiewska et al., 2017).

**Figure 5:**
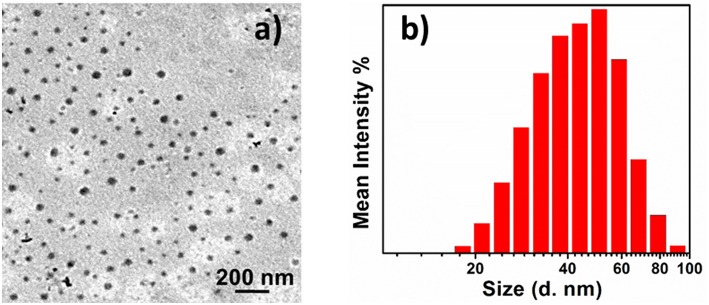
TEM image **(a)** and particle size distribution **(b)** of 0.2 mg/ml aqueous solution of **2-**EB.

The size of the structures has changed with changing the oxidation state of TANI segment in the ABA type triblock structures. Figure 5 shows the TEM image and particle size distribution of 0.2 mg/ml aqueous solution of **2-**EB. When **2-**LEB was oxidized to **2-**EB the size of the particles at concentration 0.2 mg/mL was decreased from 80 to 45 nm without changing the spherical morphology (Figure 5a). This decrease in the size of the particles is attributed to the additional amine-imine hydrogen bonding in the EB state leading to shrinkage in size of the aggregates, as has been reported previously (Kim et al., 2011). The size of these aggregates was further confirmed by DLS measurements (Figure 5b), and showed the presence of 50 ± 3 nm size of **2-**EB particles, which is correlated very well with the size observed by TEM after changing oxidation state. Ring-like morphologies for **2**-EB were also seen by TEM (Figure S3). This may be attributed to the fact that on evaporation of the solvent, these small aggregates coagulate into ring-like structures increasing the stability of the aggregates (Wang et al., 2006).

**Figure 6:**
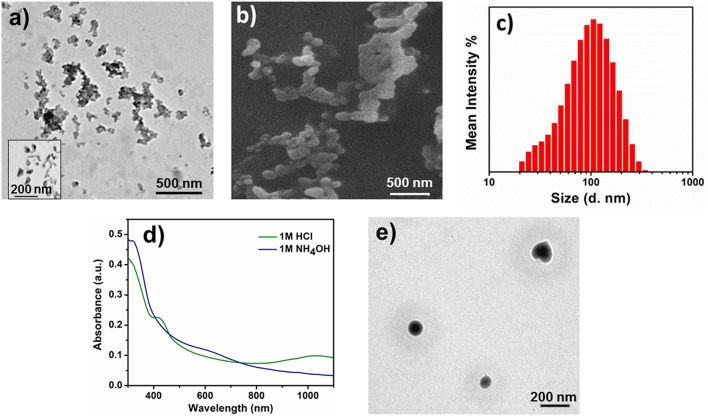
Self-assembly of doped/dedoped **2**-EB. **(a)** TEM image of **2**-ES doped with 1M HCl, **(b)** SEM image of **2**-ES, **(c)** particle size distribution curve of **2**-ES, **(d)** UV-Vis spectra of doped and dedoped **2**-EB, **(e)** TEM image of **2**-ES dedoped with 1 M NH_4_OH.

The self-assembled aggregates also showed acid/base sensitivity. When **2-**EB was doped with 1 M HCl, **2**-ES state was formed, which was confirmed by UV-Vis-NIR (Figure 3B). **2-**ES was investigated for aqueous self-assembly, and colonies of spherical aggregates of 103 nm size were observed by TEM (Figure 6a). SEM images of **2**-ES (Figure 6b) also showed such aggregated spherical objects of similar sizes. DLS analysis further confirmed the presence of particles of hydrodynamic radii of 105 ± 6 nm (Figure 6c). The increase in size of **2**-ES as compared to **2**-EB was attributed to the incorporation of acid moieties, which increased the volume of the inner core of the aggregates as well as inner repulsion of counter ions (Hu et al., 2008). Intermolecular hydrogen bonding might be the cause of the formation of dendritic colonies (Wang et al., 2012). When 2-ES was dedoped by the addition of 1 M NH_4_OH, first the visible change in color was observed from green to blue showing the change in oxidation state of the TANI segment, which was further investigated by UV-vis spectroscopy. Figure 6d shows the change in electrochemical behavior of the doped/dedoped states. 2-ES showed characteristic peaks at 305, 420, and 978 nm for benzenoid ring, localized and delocalized polarons, respectively (Shao et al., 2011; Udeh et al., 2013). Dedoping with the base caused a slight red shift from 305 to 312 nm along appearance of peak of low intensity at 580 nm for benzenoid and quinoid rings, respectively. Spherical aggregates of 70 nm size were observed by TEM image (Figure 6e). The decrease in size of the aggregates is attributed to the removal of acid moiety from the inner core of TANI segment upon dedoping as well as increased hydrophobic character of TANI (Hu et al., 2008).

## Conclusions

A novel electroactive ABA amphiphilic triblock structure **2** was successfully synthesized, and the structure and purity were confirmed by NMR and FTIR spectroscopy. UV-Vis-NIR spectroscopic and CV studies showed good electrochemical activity owing to the core TANI block. Aqueous self-assembly behavior of **2** in its various oxidation states was studied by using a range of techniques. Spherical aggregates were observed and the size of the particles was increased with increasing concentration. It was found that the size of the aggregates changed with changing the oxidation state of the core TANI moiety in **2**. On oxidation of LEB to the EB, the size of the particles was decreased. The aggregates were found to be acid/base sensitive. Doping of **2-**EB with 1 M HCl led to an increase in the size of the objects without changing the morphology, while dedoping of the amphiphiles using 1 M NH_4_OH showed a decreased in size of the particles. Such tunable self-assembly structures can be used as promising candidates for drug delivery and sensor applications.

## Data Availability

All datasets generated for this study are included in the manuscript and/or Supplementary Material.

## Author Contributions

IM performed and analyzed the experiments and wrote the manuscript. ZA and FS conceived the project. All authors contributed to the interpretation of the data and writing the manuscript.

### Conflict of Interest Statement

The authors declare that the research was conducted in the absence of any commercial or financial relationships that could be construed as a potential conflict of interest.
