# Supplementary material for: Tunable Self-Assembled Nanostructures of Electroactive PEGylated Tetra(Aniline) Based ABA Triblock Structures in Aqueous Medium
Source: Front Chem. 2019 Jul 25;7:518. doi: 10.3389/fchem.2019.00518 (PMC6669400; doi:10.3389/fchem.2019.00518)
Supplement: Supplementary file 1 [file Data_Sheet_1.pdf]

# Supplementary Material

# Tunable Self-Assembled Nanostructures of Electroactive PEGylated Tetra(Aniline) Based ABA Triblock Structures in Aqueous Medium

**Irrum Mushtaq,[a] Zareen Akhter[a,] *, and Faiz Ullah Shah[b,] ***

[a]Department of Chemistry, Quaid-i-Azam University, Islamabad 45320, Pakistan

[b]Chemistry of Interfaces, Luleå University of Technology, SE-971 87 Luleå, Sweden

[*]Corresponding authors: Email: zareenakhter@yahoo.com and faiz.ullah@ltu.se

**Content:**

Figure S-1. Critical micelle concentration (CMC) of 2-EB in water (at $\lambda_{max}$ = 312 nm)....................2

Figure S-2. Particle size distribution of 2-LEB by DLS at different concentrations: 0.2 mg/mL (a), 1.0 mg/mL (b), 5.0 mg/mL (c)..........................................................................................2

Figure S-3. TEM image of 2-EB (scale bar: 2 μm)..........................................................................3

Figure S-4. $^{1}$H NMR spectrum of $NH_2$/$NH_2$-capped TANI (1)......................................................3

Figure S-5. $^{13}$C NMR spectrum of $NH_2$/$NH_2$-capped TANI (1)....................................................4

Figure 6-S. Mass spectrum of $NH_2$/$NH_2$-capped TANI (1)..........................................................4

Figure S-7. $^{1}$H NMR of tosylated-$mPEG_{350}$.....................................................................5

Figure S-8. $^{13}$C NMR spectrum of tosylated- $mPEG_{350}$...............................................................5

Figure S-9. Mass spectrum of tosylated-$mPEG_{350}$.........................................................................6

Figure S-10. Mass spectrum of (2)..........................................................................................7

Figure S-11. Mass spectrum of 2-LEB.......................................................................................8

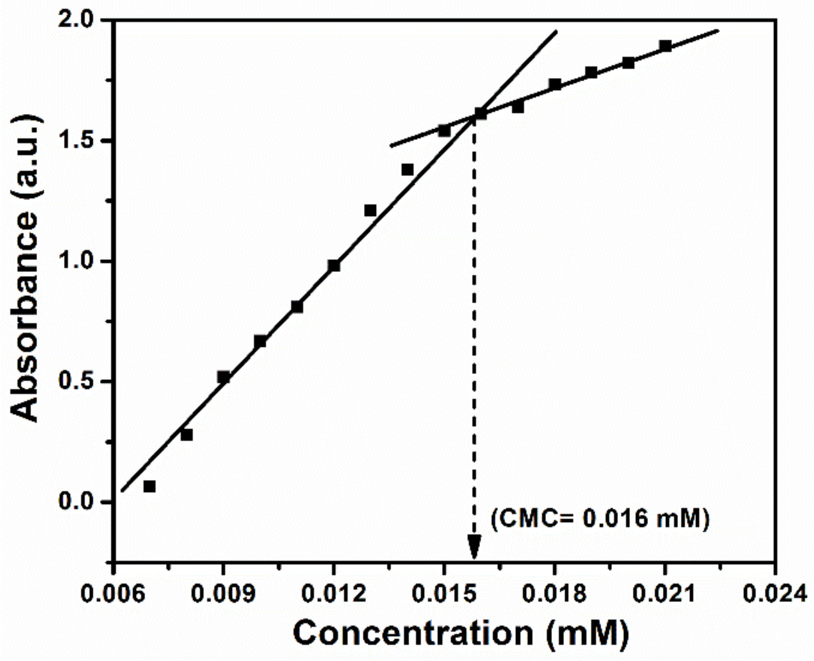


**Figure S-1**. Critical micelle concentration (CMC) of **2**-EB in water (at $\lambda_{max}$ = 312 nm)

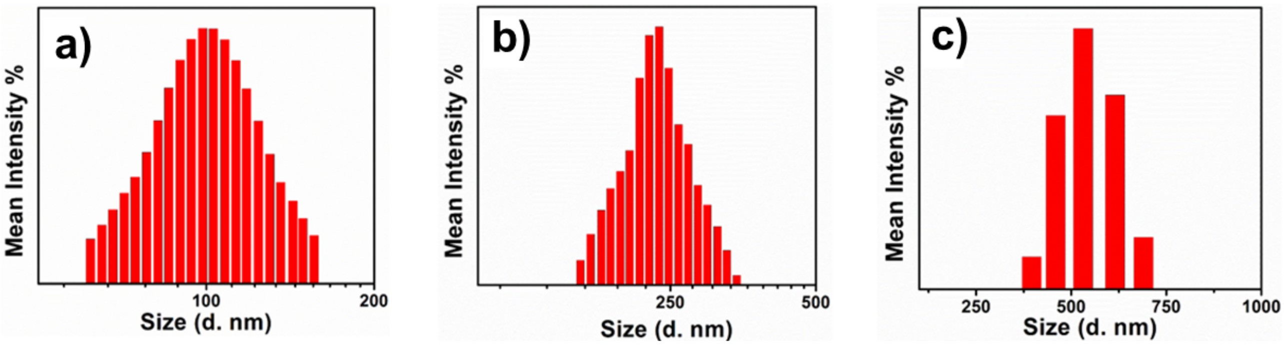


**Figure S-2**. Particle size distribution of **2**-LEB by DLS at different concentrations: 0.2 mg/mL (a), 1.0 mg/mL (b), 5.0 mg/mL (c).

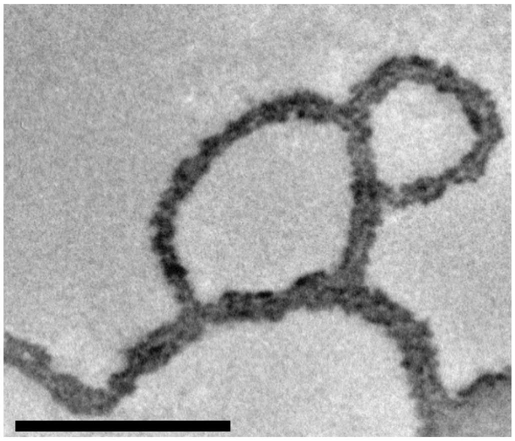

**Figure S-3**. TEM image of **2-**EB (scale bar: 2 μm)

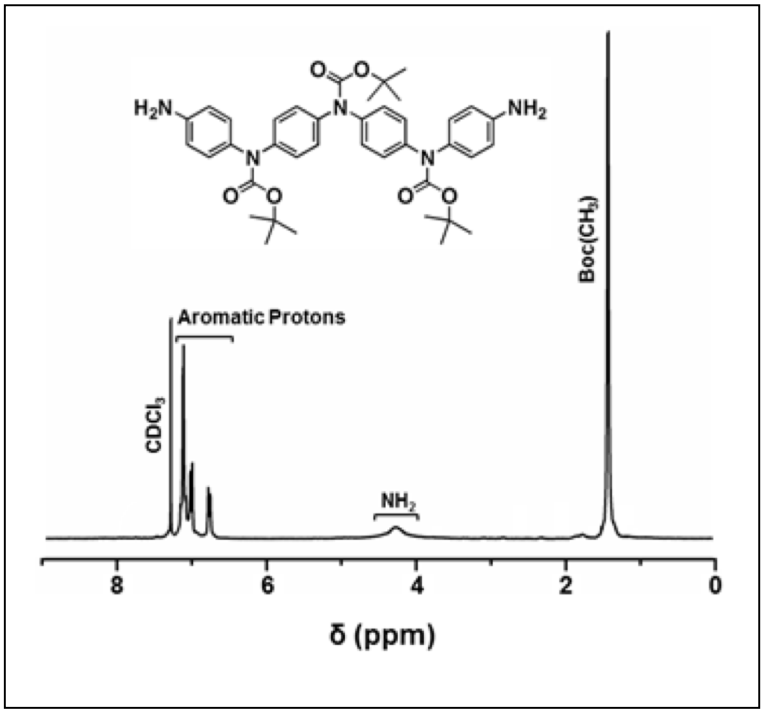


**Figure S-4.** $^1$H NMR spectrum of $NH_2/NH_2$-capped TANI (1)

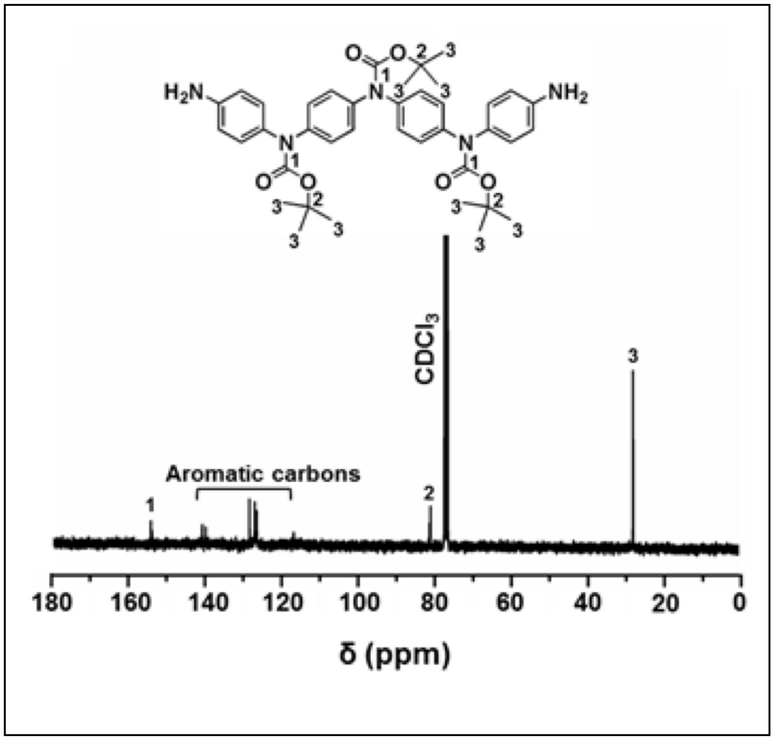


**Figure S-5.** $^{13}C$ NMR spectrum of $NH_2/NH_2$-capped TANI (1)

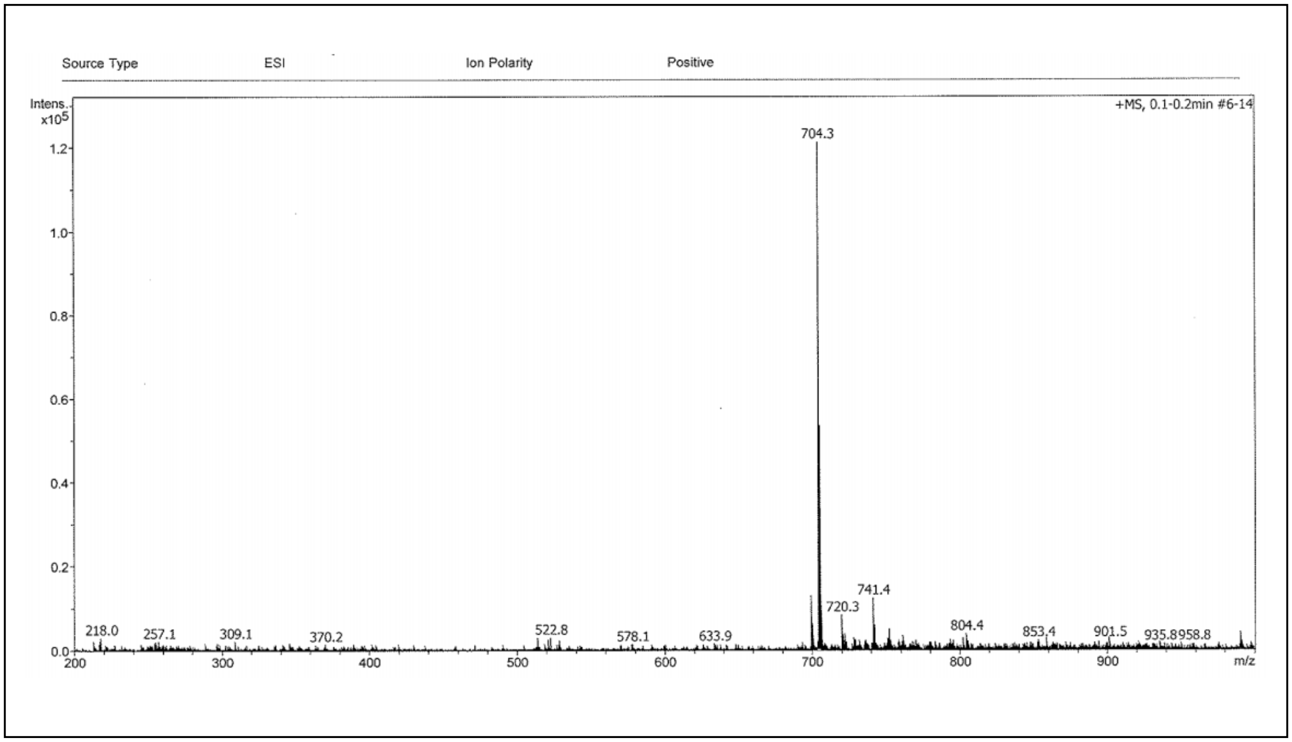


**Figure S-6.** Mass spectrum of $NH_2/NH_2$-capped TANI (1)

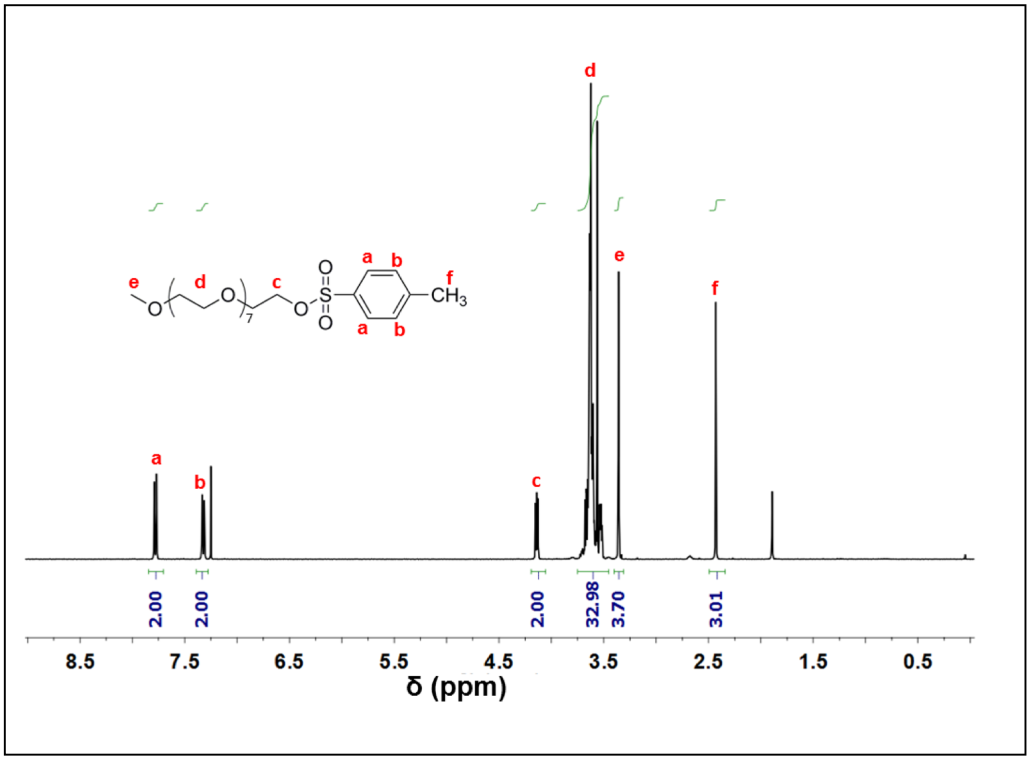


**Figure S-7.** $^{1}$H NMR of tosylated-mPEG$_{350}$

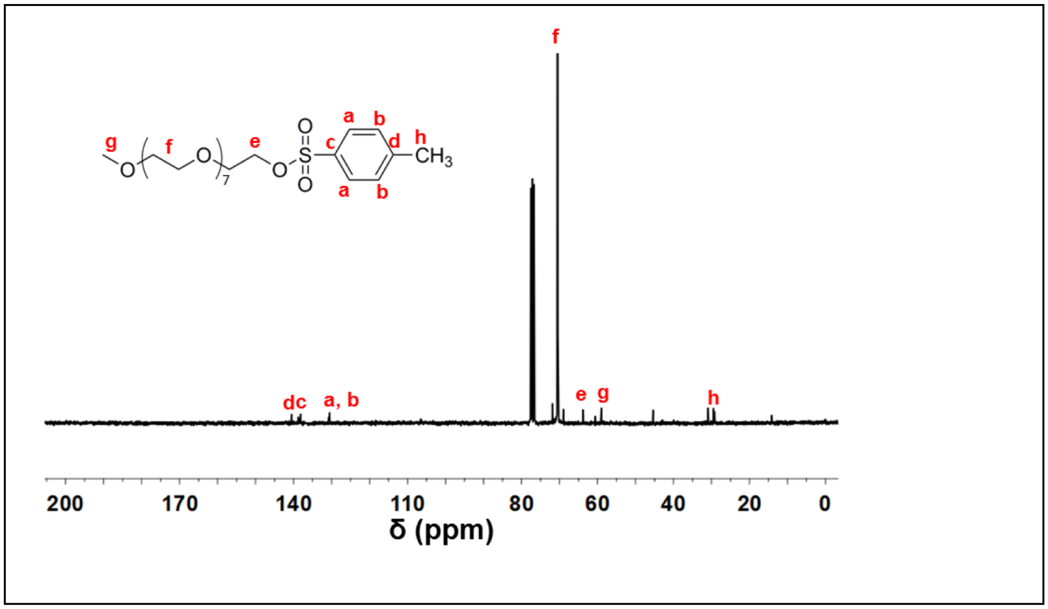


**Figure S-8.** $^{13}$C NMR spectrum of tosylated- mPEG$_{350}$

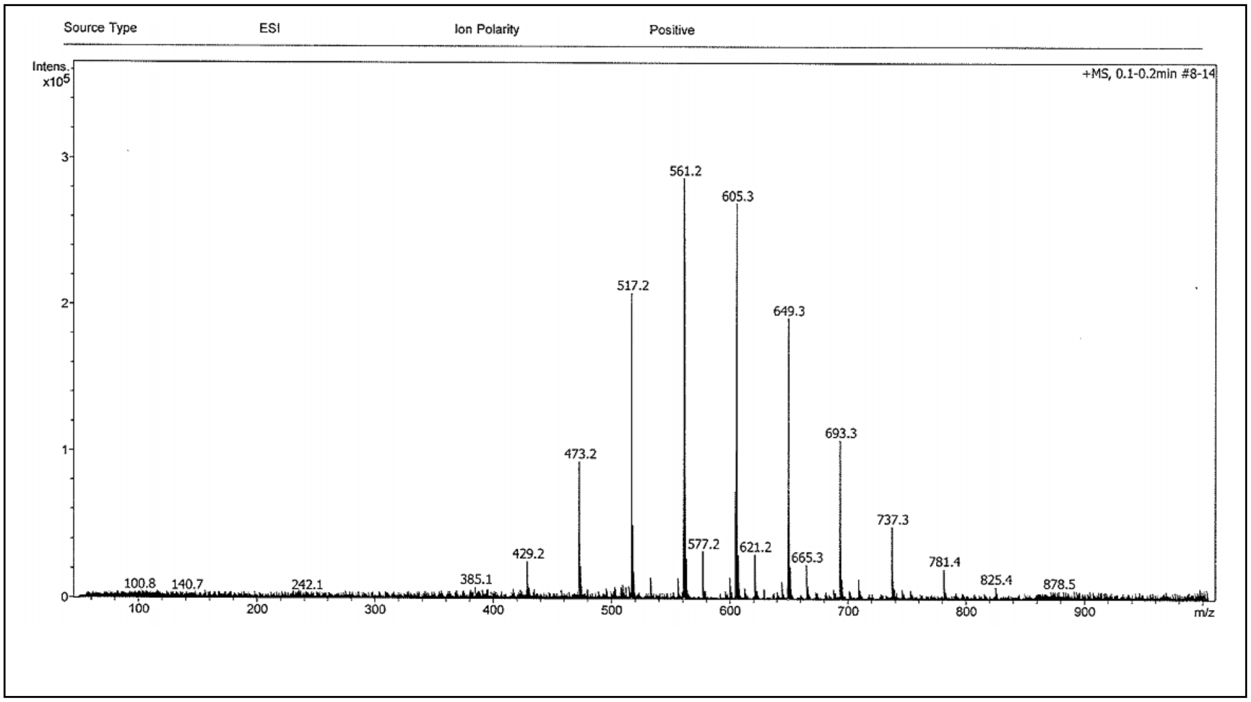


**Figure S-9.** Mass spectrum of tosylated-mPEG$_{350}$

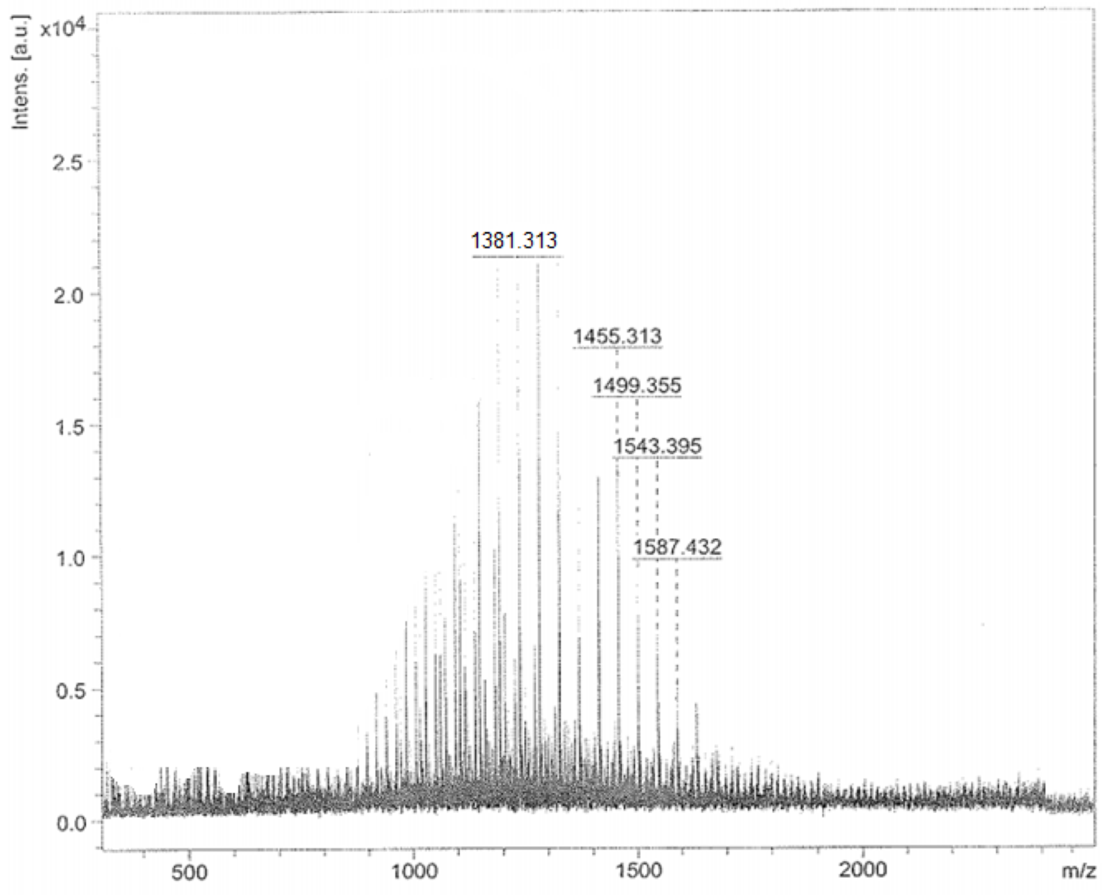


**Figure S-10.** Mass spectrum of (2)

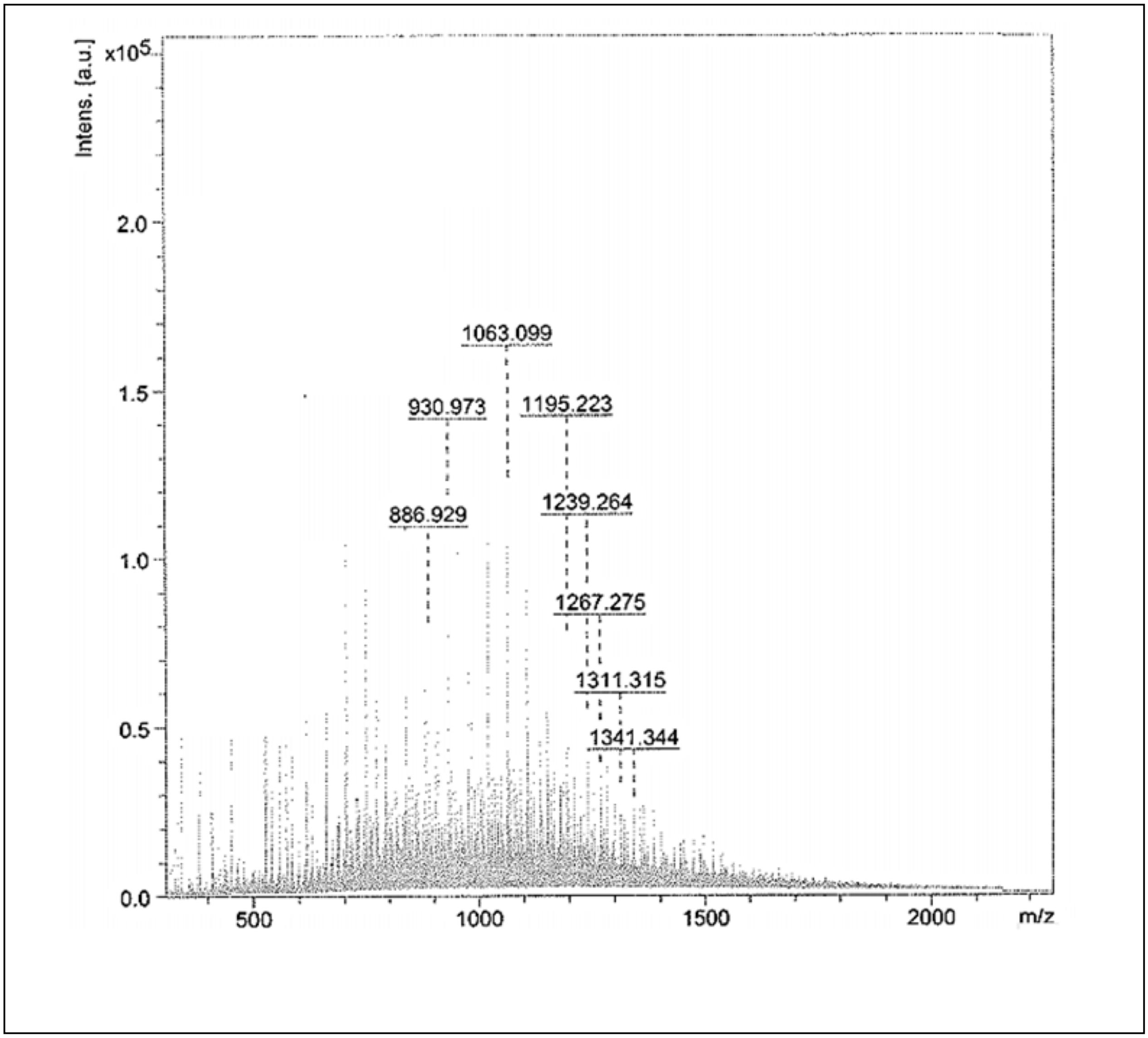


**Figure S-11.** Mass spectrum of 2-LEB
